# Candida pararugosa: First Reported Bloodstream Infection in an Adult

**DOI:** 10.7759/cureus.1283

**Published:** 2017-05-29

**Authors:** Guy El Helou, Elizabeth Palavecino

**Affiliations:** 1 Infectious Disease, Wake Forest Baptist Medical Center; 2 Clinical Pathology, Wake Forest Baptist Medical Center

**Keywords:** candida pararugosa, candidemia, blood-stream infection

## Abstract

*Candida pararugosa* is a yeast that has been previously isolated in various human specimens. The first reported isolation was from human feces in 1998, with subsequent reports of positive cultures from the oral cavity where it was thought to represent colonization rather than true infection. Though it has been isolated from other human sites, its clinical significance and manifestations are poorly characterized. We report the case of a 39-year-old woman on parenteral hyperalimentation who developed post abdominal surgery sepsis and surgical wound necrotizing fasciitis. *Candida pararugosa* was isolated from two different blood cultures and the patient’s clinical status improved after initiation of therapy with micafungin. Though it was not clear whether sepsis was driven by the candidemia or the necrotizing fasciitis or both, this report appears to be the first case of *Candida pararugosa* bloodstream infection described in an adult.

## Introduction

*Candida pararugosa* (*C. pararugosa*) was first described in the medical literature in 1999 by Nakase, et al. [1]. It was isolated from human feces and believed to be a colonizer of the gastrointestinal (GI) tract. Since then, additional reports in the literature have established this yeast as an occasional colonizer of the oral cavity as well [2-3]. Though subsequently isolated from various human anatomical sites and specimens [4], no case reports in the medical literature are available to correlate its isolation with clinical disease. To the best of our knowledge, this report represents the first case of *C. pararugosa *isolated from blood in an adult.

## Case presentation

A 39-year-old female underwent duodenal switch with biliopancreatic diversion surgery for morbid obesity in July 2016. Her post-operative course was complicated by delayed emptying of the stomach and duodeno-ileal anastomotic stricture treated by three endoscopic dilatations leading to severe weight loss and failure to thrive. A peripherally inserted central catheter (PICC) was placed in January 2017 and she was started on total parenteral nutrition (TPN). In March 2017, she underwent duodenal switch revision surgery with J-tube placement. This procedure was complicated by sepsis and necrotizing fasciitis of the surgical wound requiring broad-spectrum antibiotics (vancomycin and piperacillin/tazobactam) and repetitive incision and debridement (I&D). Despite apparent good source control, she continued to exhibit fevers and had hypotension. Central and peripheral blood cultures (BC) were obtained and her piperacillin/tazobactam was changed to meropenem. Two days later, both of her BC grew yeast and micafungin 100 mg IV daily was started and the PICC was removed. After receiving micafungin for 48 hours, her fevers resolved and her hypotension improved. The yeast was finally identified as *C. pararugosa *with MALDI-TOF, though no GenBank/MALDI BioTyper accession could be generated. The patient was continued on micafungin as her isolate had minimal inhibitory concentration (MIC) of 8 μg/ml to fluconazole. Subsequent BC were negative and therapy was continued for two weeks after PICC removal. Dilated eye exam by ophthalmology found no evidence of ocular involvement.

## Discussion

*C. pararugosa* is now recognized as a distinct *Candida* species different from *Candida rugosa spp complex *[5]. Though morphologically different, misidentification of this species as *Candida rugosa* (*C. rugosa*) is common when phenotypic characteristics are analyzed [5]. *C. pararugosa* has been isolated from various environmental sources and foods [6-8] and has been mentioned as a potential probiotic for its ability to resist toxic effects of bile and gastric secretions [9]. Human isolations of this yeast were initially from feces and the oral cavity [1-3], suggesting a potential role as a colonizer of the GI tract. Newer molecular studies have facilitated the identification of *C. pararugosa *from human bronchial washes, blood, urine, and vagina [4, 10]. Limited clinical data on the patients from whom the positive cultures were obtained are provided. However, there is a reference to a 6-month-old male with intrauterine growth restriction from Qatar treated with liposomal amphotericin B who died from *C. pararugosa *bloodstream infection as reported by Taj-Aldeen, et al. [10]. *C. pararugosa *has also been implicated as a potential pathogen in fingernail onychomycosis. The pathogenic role that this yeast actually plays in human disease is still uncertain. *Candida spp.* isolated from blood cultures are usually associated with high likelihood of invasive disease and high mortality. In our patient, micafungin was initiated before speciation of the yeast occurred, with subsequent clinical and hemodynamic improvement. It is possible that this improvement was the delayed effect of necrotizing fasciitis control, but the timeline of events was more compatible with control of candidemia. Our isolate’s MICs were consistent with previously reported high MIC to fluconazole and low MIC to amphotericin [4-5, 10]. MICs for the *C. pararugosa *strain isolated from our patient are listed in Table 1. Though no susceptibility breakpoints are established for *C. pararugosa*, we decided to continue with micafungin as the MIC for fluconazole was high when compared to other *Candida spp. *with established breakpoints.

**Table 1:** Candida pararugosa minimal inhibitory concentration (MIC) (ug/mL) compared to Candida albicans breakpoints ^a^​Described in the case ^b^*Candida albicans *breakpoint from CLSI M27-S4, 2012

Anti-fungal agent	*Candida pararugosa *MIC^a^	*Candida albicans *resistance breakpoint^b^
5-Fluorocytosine	= 0.12	
Amphotericin B	= 1	
Fluconazole	= 8	>= 8
Itraconazole	= 0.12	
Micafungin	= 0.06	>= 1

## Conclusions

The goal of this report is to raise clinical awareness of heretofore uncommon pathogens detected by newer techniques such as MALDI-TOF or molecular methods. Our patient represents the first detailed clinical report of *C. pararugosa *infection in an adult. The only previously reported case of bloodstream infection with *C. pararugosa *was in a child who* *died despite treatment with intravenous amphotericin B. Our isolate as well as previously reported isolates exhibit high MICs to fluconazole. Though no susceptibility breakpoints are available, the generally high MICs to fluconazole warrant consideration of therapy with micafungin or another echinocandin.

## References

[1] Suzuki M, Suh S-O, Sugita T (1999). A phylogenetic study on galactose-containing Candida species based on 18S ribosomal DNA sequences. J Gen Appl Microbiol.

[2] Giammanco GM, Melilli D, Pizzo G (2004). Candida pararugosa isolation from the oral cavity of an Italian denture wearer. Res Microbiol.

[3] Nakagawa Y, Robert V, Kawarazaki J (2004). Recurrent isolation of an uncommon yeast, Candida pararugosa, from a sarcoma patient. Med Mycol.

[4] Paredes K, Sutton DA, Cano J (2012). Molecular identification and antifungal susceptibility testing of clinical isolates of the Candida rugosa species complex and proposal of the new species Candida neorugosa. J Clin Microbiol.

[5] Padovan AC, Melo AS, Colombo AL (2013). Systematic review and new insights into the molecular characterization of the Candida rugosa species complex. Fungal Genet Biol.

[6] Jensen SL, Umiker NL, Arneborg N (2009). Identification and characterization of Dekkera bruxellensis, Candida pararugosa, and Pichia guilliermondii isolated from commercial red wines. Food Microbiol.

[7] Gori K, Bjørklund MK, Canibe N (2011). Occurrence and identification of yeast species in fermented liquid feed for piglets. Microb Ecol.

[8] Callon C, Duthoit F, Delbès C (2007). Stability of microbial communities in goat milk during a lactation year: molecular approaches. Syst Appl Microbiol.

[9] Pennacchia C, Blaiotta G, Pepe O (2008). Isolation of Saccharomyces cerevisiae strains from different food matrices and their preliminary selection for a potential use as probiotics. J Appl Microbiol.

[10] Taj-Aldeen SJ, AbdulWahab A, Kolecka A (2014). Uncommon opportunistic yeast bloodstream infections from Qatar. Med Mycol.

